# Supplementary material for: Childhood Maltreatment and Its Interaction with Hypothalamic–Pituitary–Adrenal Axis Activity and the Remission Status of Major Depression: Effects on Functionality and Quality of Life
Source: Brain Sci. 2021 Apr 13;11(4):495. doi: 10.3390/brainsci11040495 (PMC8069655; doi:10.3390/brainsci11040495)
Supplement: Supplementary file 1 [file brainsci-11-00495-s001.zip › TableS1.pdf]

**Table S1.** Sample characteristics, clinical variables and cortisol measures by study groups (adapted from Salvat-Pujol et al., 2017)[1].

| | HC n=97 | Remitted MDD n=44 | Non-remitted MDD n=53 | Statistics (ANOVA / T-test / $\chi^2$) |
|---|---|---|---|---|
| Age (years) | 56.59 (11.86) | 62.09 (11.69) | 57.89 (11.48) | F(193)=3.353, p=0.037[a] |
| Female gender, n (%) | 64 (66.0) | 29 (65.9) | 39 (73.6) | $\chi^2$=1.031, p= 0.597 |
| Education (years) | 12.10 (4.06) | 8.50 (4.29) | 9.15 (4.04) | F(193)=15.477, p<0.001[a,b] |
| BMI (kg/m2) | 26.75 (4.87) | 27.88 (4.28) | 29.72 (5.33) | F(192)=6.329, p=0.002[b] |
| PSQI | 5.19 (3.31) | 6.51 (3.44) | 11.53 (4.70) | F(167)=42.017, p<0.001[b,c] |
| Substance use | | | | |
| Smoking, n (%) | 16 (16.5) | 11 (25.0) | 14 (26.4) | $\chi^2$=2.638, p= 0.267 |
| Tobacco consumption (cigarettes/day) | 2.35 (7.40) | 3.20 (6.67) | 3.96 (8.73) | F(193)=0.788, p=0.456 |
| Daily alcohol intake, n (%) | 31 (32.0) | 16 (36.4) | 10 (18.9) | $\chi^2$=4.423, p=0.110 |
| Daily alcohol intake (g/day) | 2.52 (6.10) | 1.92 (3.59) | 1.37 (3.92) | F(173)=0.821, p=0.442 |
| Clinical variables | | | | |
| STAI - state | 11.10 (6.69) | 17.57 (10.76) | 34.17 (12.91) | F(193)=97.583, p<0.001[a,b,c] |
| STAI - trait | 15.73 (9.08) | 20.45 (11.76) | 38.67 (10.33) | F(178)=83.239, p<0.001[a,b,c] |
| HDRS | 0.71 (1.14) | 2.48 (2.32) | 17.57 (6.81) | F(193)=357.048, p<0.001[a,b,c] |
| Suicide attempts, n (%) | 0 (0) | 9 (20.5) | 15 (28.3) | $\chi^2$=28.754, p<0.001 |
| Suicide attempts | 0 (0) | 0.31 (0.92) | 0.45 (0.93) | F(191)=9.156, p<0.001[a,b] |
| Age of onset (years)* | NA | 42.38 (13.02) | 41.61 (14.60) | t(89)=0.263, p=0.793 |
| Melancholic symptoms, n (%)* | NA | 35 (79.5) | 46 (86.8) | $\chi^2$=1.699, p=0.428 |
| Atypical symptoms, n (%)* | NA | 0 (0) | 3 (5.7) | $\chi^2$=2.512, p=0.250 |
| Number of depressive episodes* | NA | 3.89 (2.89) | 3.71 (2.19) | t(94)=0.337, p=0.737 |
| Number of hospitalizations* | NA | 0.60 (1.22) | 1.04 (1.97) | t(88)=-1.237, p=0.197 |
| Cortisol measures | | | | |
| Waking cortisol (nmol/L) (T1) | 16.63 (8.16) | 15.83 (7.86) | 17.83 (10.95) | F(173)=0.643, p=0.527 |
| 30 min post-awakening cortisol (nmol/L) (T2) | 22.90 (10.70) | 22.43 (12.45) | 22.00 (12.36) | F(174)=0.266, p=0.767 |
| 60 min post-awakening cortisol (nmol/L) (T3) | 17.17 (8.76) | 17.22 (11.05) | 19.08 (11.07) | F(174)=0.500, p=0.607 |
| 10-h cortisol (nmol/L) (T4) | 11.53 (10.72) | 11.41 (8.10) | 13.50 (9.74) | F(174)=0.949, p=0.389 |
| 23-h cortisol (nmol/L) (T5) | 3.07 (2.41) | 3.34 (2.74) | 3.89 (2.28) | F(180)=3.344, p=0.038[b] |
| 10-h post-DXM cortisol (nmol/L) (T6) | 5.02 (5.37) | 6.75 (8.04) | 6.89 (7.85) | F(179)=2.359, p=0.097 |
| CAR† | 19.67 (54.08) | 28.40 (61.56) | 14.08 (56.28) | F(170)=0.656, p=0.520 |
| DSTR‡ | 9.62 (19.69) | 3.66 (4.13) | 6.62 (10.90) | F(179)=2.180, p=0.116 |
| Diurnal cortisol slope | -0.15 (0.11) | -0.13 (0.14) | -0.15 (0.11) | F(171)=0.214, p=0.808 |

[1]Salvat-Pujol N, et al. 2017. Hypothalamic-pituitary-adrenal axis activity and cognition in major depression: The role of remission status. Psychoneuroendocrinology 76:38–48. Abbreviations: HC, healthy controls; MDD, major depressive disorder; BMI, body mass index; PSQI, Pittsburgh Sleep Quality Index; STAI: State-Trait Anxiety Inventory; HDRS, Hamilton Depression Rating Scale; DXM, dexamethasone; CAR, cortisol awakening response calculated to the increase; DSTR, dexamethasone suppression test ratio.

All variables presented in mean (SD) or n (%).

Cortisol raw scores and untransformed DSTR are shown, outliers excluded. P values calculated upon transformed cortisol and DSTR values, outliers excluded.

†CAR were calculated with transformed cortisol values.

‡DSTR= 10-h cortisol/10-h post-DXM cortisol.

NA: not applicable.

* Statistical analyses performed using only Remitted MDD and Non-remitted MDD groups.

[a] Significant ANOVA post hoc analyses (comparison between groups) with Bonferroni correction: HC vs. remitted MDD.

[b] Significant ANOVA post hoc analyses (comparison between groups) with Bonferroni correction: HC vs. non-remitted MDD.

[c] Significant ANOVA post hoc analyses (comparison between groups) with Bonferroni correction: remitted MDD vs. non-remitted MDD.
