# Supplementary material for: Childhood Maltreatment and Its Interaction with Hypothalamic–Pituitary–Adrenal Axis Activity and the Remission Status of Major Depression: Effects on Functionality and Quality of Life
Source: Brain Sci. 2021 Apr 13;11(4):495. doi: 10.3390/brainsci11040495 (PMC8069655; doi:10.3390/brainsci11040495)
Supplement: Supplementary file 1 [file brainsci-11-00495-s001.zip › TableS2.pdf]

Table S2. Results of partial correlation analyses in healthy controls.

	CTQ - emotional abuse	CTQ - physical abuse	CTQ - sexual abuse	CTQ - emotional neglect	CTQ - physical neglect	CTQ - total score	STAI - state	STAI - trait	Waking cortisol (nmol/L)	†CAR	‡DSTR	Diurnal cortisol slope	SASS	EQ-5D – Index	EQ-5D – VAS
CTQ - emotional abuse	1	0.585**	0.240*	0.442**	0.468**	0.806*	-0.058	0.022	0.011	0.086	0.009	-0.067	-0.116	-0.079	0.130
CTQ - physical abuse	0.585**	1	0.012	0.190	0.199	0.530*	-0.054	-0.036	-0.050	0.111	-0.081	-0.074	-0.064	-0.020	0.038
CTQ - sexual abuse	0.240*	0.012	1	0.194	0.117	0.359*	-0.008	-0.022	0.028	0.106	-0.101	0.079	0.040	0.049	0.024
CTQ - emotional neglect	0.442**	0.190	0.194	1	0.582**	0.835*	-0.093	0.056	-0.065	-0.067	-0.142	0.192	0.011	0.050	0.081
CTQ - physical neglect	0.468**	0.199	0.117	0.582**	1	0.723*	-0.280*	-0.073	-0.052	-0.039	-0.038	0.122	0.052	0.197	0.177
CTQ - total score	0.806**	0.530**	0.359**	0.835**	0.723**	1	-0.140	0.008	-0.045	0.025	-0.107	0.097	-0.028	0.046	0.134
STAI - state	-0.058	-0.054	-0.008	-0.093	-0.280*	-0.140	1	0.656**	0.088	-0.076	0.149	-0.133	-0.173	-0.410**	-0.241*
STAI - trait	0.022	-0.036	-0.022	0.056	-0.073	0.008	0.656**	1	0.008	-0.071	0.187	-0.172	-0.251*	-0.429**	-0.332**
Waking cortisol	0.011	-0.050	0.028	-0.065	-0.052	-0.045	0.088	0.008	1	-0.565**	-0.092	-0.087	-0.184	0.107	0.027
†CAR	0.086	0.111	0.106	-0.067	-0.039	0.025	-0.076	-0.071	-0.565**	1	0.334**	-0.252*	0.009	0.104	0.075
‡DSTR	0.009	-0.081	-0.101	-0.142	-0.038	-0.107	0.149	0.187	-0.092	0.334**	1	-0.393**	-0.008	0.048	-0.065
Diurnal cortisol slope	-0.067	-0.074	0.079	0.192	0.122	0.097	-0.133	-0.172	-0.087	-0.252*	-0.393**	1	0.135	-0.004	0.078
SASS	-0.116	-0.064	0.040	0.011	0.052	-0.028	-0.173	-0.251*	-0.184	0.009	-0.008	0.135	1	0.241*	0.187
EQ-5D – Index	-0.079	-0.020	0.049	0.050	0.197	0.046	-0.410**	-0.429**	0.107	0.104	0.048	-0.004	0.241*	1	0.530**
EQ-5D – VAS	0.130	0.038	0.024	0.081	0.177	0.134	-0.241*	-0.332**	0.027	0.075	-0.065	0.078	0.187	0.530**	1

Partial correlation analyses adjusted by gender, age, and years of education.
Pearson’s correlation coefficients are shown. Significant results are marked with asterisks (* $p<0.05$; ** $p<0.01$).
Abbreviations: CTQ, Childhood Trauma Questionnaire; STAI: State-Trait Anxiety Inventory; CAR, cortisol awakening response calculated to the increase; DSTR, dexamethasone suppression test ratio; SASS, Social Adaptation Self-evaluation Scale; EQ-5D, Euro Quality of Life 5-dimensions questionnaire; VAS, Visual Analogue Scale.
Correlations between cortisol values calculated from transformed variables, outliers excluded.
