# Supplementary material for: Childhood Maltreatment and Its Interaction with Hypothalamic–Pituitary–Adrenal Axis Activity and the Remission Status of Major Depression: Effects on Functionality and Quality of Life
Source: Brain Sci. 2021 Apr 13;11(4):495. doi: 10.3390/brainsci11040495 (PMC8069655; doi:10.3390/brainsci11040495)
Supplement: Supplementary file 1 [file brainsci-11-00495-s001.zip › TableS3.pdf]

Table S3. Results of partial correlation analyses in patients with remitted MDD.

	CTQ - emotional abuse	CTQ - physical abuse	CTQ - sexual abuse	CTQ - emotional neglect	CTQ - physical neglect	CTQ - total score	STAI - state	STAI - trait	Waking cortisol (nmol/L)	†CAR	‡DSTR	Diurnal cortisol slope	SASS	EQ-5D – Index	EQ-5D – VAS
CTQ - emotional abuse	1	0.630**	0.542**	0.238	-0.146	0.791*	0.351	0.314	0.144	-0.190	0.031	-0.170	-0.195	-0.386	-0.580**
CTQ - physical abuse	0.630**	1	0.116	-0.102	-0.129	0.461*	0.221	0.145	0.200	0.206	-0.119	-0.052	-0.107	-0.338	-0.326
CTQ - sexual abuse	0.542**	0.116	1	0.102	0.064	0.553*	0.282	0.078	-0.002	-0.086	0.046	-0.125	0.166	-0.144	-0.234
CTQ - emotional neglect	0.238	-0.102	0.102	1	0.252	0.693*	-0.112	0.144	-0.311	-0.068	0.168	-0.236	0.082	-0.127	-0.104
CTQ - physical neglect	-0.146	-0.129	0.064	0.252	1	0.261	0.188	-0.095	0.053	-0.052	0.206	-0.086	0.189	-0.268	0.099
CTQ - total score	0.791**	0.461*	0.553**	0.693**	0.261	1	0.239	0.241	-0.053	-0.086	0.125	-0.258	0.019	-0.399	-0.407*
STAI - state	0.351	0.221	0.282	-0.112	0.188	0.239	1	0.384	-0.056	0.157	0.311	0.050	-0.177	-0.402	-0.254
STAI - trait	0.314	0.145	0.078	0.144	-0.095	0.241	0.384	1	0.079	0.207	0.289	-0.325	-0.216	-0.495*	-0.382
Waking cortisol	0.144	0.200	-0.002	-0.311	0.053	-0.053	-0.056	0.079	1	-0.323	0.053	-0.182	0.136	-0.167	-0.044
†CAR	-0.190	0.206	-0.086	-0.068	-0.052	-0.086	0.157	0.207	-0.323	1	-0.066	-0.198	-0.060	-0.150	-0.179
‡DSTR	0.031	-0.119	0.046	0.168	0.206	0.125	0.311	0.289	0.053	-0.066	1	-0.395	-0.134	-0.216	-0.070
Diurnal cortisol slope	-0.170	-0.052	-0.125	-0.236	-0.086	-0.258	0.050	-0.325	-0.182	-0.198	-0.395	1	0.030	0.457*	0.433*
SASS	-0.195	-0.107	0.166	0.082	0.189	0.019	-0.177	-0.216	0.136	-0.060	-0.134	0.030	1	0.154	0.400
EQ-5D – Index	-0.386	-0.338	-0.144	-0.127	-0.268	-0.399	-0.402	-0.495*	-0.167	-0.150	-0.216	0.457*	0.154	1	0.349
EQ-5D – VAS	-0.580**	-0.326	-0.234	-0.104	0.099	-	-0.254	-0.382	-0.044	-0.179	-0.070	0.433*	0.400	0.349	1

Partial correlation analyses adjusted by gender, age, and years of education.

Pearson's correlation coefficients are shown. Significant results are marked with asterisks (* p<0.05; ** p<0.01).

Abbreviations: MDD, major depressive disorder; CTQ, Childhood Trauma Questionnaire; STAI: State-Trait Anxiety Inventory; CAR, cortisol awakening response calculated to the increase; DSTR, dexamethasone suppression test ratio; SASS, Social Adaptation Self-evaluation Scale; EQ-5D, Euro Quality of Life 5-dimensions questionnaire; VAS, Visual Analogue Scale.

Correlations between cortisol values calculated from transformed variables, outliers excluded.
