# Supplementary material for: Childhood Maltreatment and Its Interaction with Hypothalamic–Pituitary–Adrenal Axis Activity and the Remission Status of Major Depression: Effects on Functionality and Quality of Life
Source: Brain Sci. 2021 Apr 13;11(4):495. doi: 10.3390/brainsci11040495 (PMC8069655; doi:10.3390/brainsci11040495)
Supplement: Supplementary file 1 [file brainsci-11-00495-s001.zip › TableS4.pdf]

Table S4. Results of partial correlation analyses in patients with non-remitted MDD.

	CTQ - emotional abuse	CTQ - physical abuse	CTQ - sexual abuse	CTQ - emotional neglect	CTQ - physical neglect	CTQ - total score	STAI - state	STAI - trait	Waking cortisol (nmol/L)	†CAR	‡DSTR	Diurnal cortisol slope	SASS	EQ-5D – Index	EQ-5D – VAS
CTQ - emotional abuse	1	0.697**	0.498**	0.570**	0.725**	0.899*	-0.144	0.168	-0.207	-0.118	-0.107	0.358	0.198	-0.196	-0.019
CTQ - physical abuse	0.697**	1	0.632**	0.277	0.499**	0.801*	0.161	0.139	-0.053	0.037	-0.043	0.199	0.089	-0.262	-0.144
CTQ - sexual abuse	0.498**	0.632**	1	0.215	0.590**	0.729*	0.131	0.316	-0.001	0.171	0.141	0.010	0.141	-0.347	-0.259
CTQ - emotional neglect	0.570**	0.277	0.215	1	0.595**	0.673*	0.060	0.292	0.197	-0.398*	-0.015	0.110	-0.149	-0.154	-0.112
CTQ - physical neglect	0.725**	0.499**	0.590**	0.595**	1	0.842*	0.012	0.343	-0.155	-0.095	-0.096	0.223	0.111	-0.160	-0.198
CTQ - total score	0.899**	0.801**	0.729**	0.673**	0.842**	1	0.048	0.305	-0.057	-0.107	-0.033	0.237	0.101	-0.283	-0.173
STAI - state	-0.144	0.161	0.131	0.060	0.012	0.048	1	0.426*	0.176	-0.046	-0.180	-0.053	-0.468**	-0.369*	-0.576**
STAI - trait	0.168	0.139	0.316	0.292	0.343	0.305	0.426*	1	0.038	-0.103	0.202	-0.165	-0.343	-0.508**	-0.495**
Waking cortisol	-0.207	-0.053	-0.001	0.197	-0.155	-0.057	0.176	0.038	1	-0.468**	0.284	-0.150	-0.143	0.135	0.023
†CAR	-0.118	0.037	0.171	-0.398*	-0.095	-0.107	-0.046	-0.103	-0.468**	1	-0.063	-0.191	0.251	-0.036	-0.121
‡DSTR	-0.107	-0.043	0.141	-0.015	-0.096	-0.033	-0.180	0.202	0.284	-0.063	1	-0.252	0.291	-0.012	0.097
Diurnal cortisol slope	0.358	0.199	0.010	0.110	0.223	0.237	-0.053	-0.165	-0.150	-0.191	-0.252	1	0.240	0.121	0.230
SASS	0.198	0.089	0.141	-0.149	0.111	0.101	-0.468**	-0.343	-0.143	0.251	0.291	0.240	1	0.421*	0.558**
EQ-5D – Index	-0.196	-0.262	-0.347	-0.154	-0.160	-0.283	-0.369*	-0.508**	0.135	-0.036	-0.012	0.121	0.421*	1	0.704**
EQ-5D – VAS	-0.019	-0.144	-0.259	-0.112	-0.198	-0.173	-0.576**	-0.495**	0.023	-0.121	0.097	0.230	0.558**	0.704**	1

Partial correlation analyses adjusted by gender, age, and years of education.

Pearson's correlation coefficients are shown. Significant results are marked with asterisks (* $p<0.05$; ** $p<0.01$).

Abbreviations: MDD, major depressive disorder; CTQ, Childhood Trauma Questionnaire; STAI: State-Trait Anxiety Inventory; CAR, cortisol awakening response calculated to the increase; DSTR, dexamethasone suppression test ratio; SASS, Social Adaptation Self-evaluation Scale; EQ-5D, Euro Quality of Life 5-dimensions questionnaire; VAS, Visual Analogue Scale.

Correlations between cortisol values calculated from transformed variables, outliers excluded.
