# Supplementary material for: Childhood Maltreatment and Its Interaction with Hypothalamic–Pituitary–Adrenal Axis Activity and the Remission Status of Major Depression: Effects on Functionality and Quality of Life
Source: Brain Sci. 2021 Apr 13;11(4):495. doi: 10.3390/brainsci11040495 (PMC8069655; doi:10.3390/brainsci11040495)
Supplement: Supplementary file 1 [file brainsci-11-00495-s001.zip › TableS5.pdf]

Table S5. Results of multiple linear regression analyses exploring the association of childhood maltreatment, HPA axis function, and MDD remission status with disability (SASS) in MDD patients (n=97).

	Model 1 $R^2 = 0.094$		Model 2 $R^2 = 0.517$		Model 3 $R^2 = 0.611$		Model 4 $R^2 = 0.623$		Final model $R^2 = 0.740$	
	β	p	β	p	β	p	β	p	β	P
CTQ- emotional abuse	-0.033	0.873	0.171	0.293	0.153	0.398	0.166	0.414	0.158	0.365
CTQ- physical abuse	-0.013	0.942	-0.119	0.380	-0.157	0.314	-0.163	0.343	-0.372	0.024
CTQ- sexual abuse	0.095	0.520	0.034	0.759	0.034	0.771	0.026	0.840	-0.079	0.496
CTQ- emotional neglect	-0.333	0.064	-0.095	0.498	-0.081	0.573	-0.073	0.649	0.636	0.013
CTQ- physical neglect	0.114	0.486	0.004	0.973	0.003	0.982	0.001	0.993	-0.108	0.380
STAI - state			-0.459	0.002	-0.269	0.104	-0.297	0.090	-0.312	0.039
STAI - trait			-0.312	0.060	-0.256	0.154	-0.257	0.211	-0.462	0.015
Gender					0.085	0.474	0.075	0.552	0.050	0.648
Age					-0.061	0.678	-0.067	0.661	-0.120	0.387
Years of education					-0.057	0.653	-0.091	0.506	-0.020	0.864
BMI					-0.242	0.050	-0.213	0.130	-0.203	0.100
Tobacco consumption (cig/day)					-0.091	0.446	-0.104	0.422	-0.338	0.013
Remitted MDD					0.121	0.415	0.131	0.408	0.159	0.256
PSQI					-0.152	0.330	-0.113	0.522	0.155	0.367
Waking cortisol							0.068	0.637	0.175	0.188
CAR							0.100	0.521	0.239	0.094
DSTR							0.056	0.662	0.031	0.777
Diurnal cortisol slope							0.122	0.349	-10.135	0.011
Diurnal cortisol slope x CTQ- emotional neglect									10.462	0.004
DSTR x Remitted MDD									0.232	0.028

SASS score was considered as the dependent variable.

β: standardized beta coefficient.

Abbreviations: MDD, major depressive disorder; SASS, Social Adaptation Self-evaluation Scale; CTQ, Childhood Trauma Questionnaire; STAI: State-Trait Anxiety Inventory; BMI, body mass index; PSQI, Pittsburgh Sleep Quality Index; CAR, cortisol awakening response calculated to the increase in cortisol; DSTR, dexamethasone suppression test ratio.

Analyses performed using transformed cortisol variables, outliers excluded.

Non-significant interaction terms were excluded in the final equation.
