# Supplementary material for: Childhood Maltreatment and Its Interaction with Hypothalamic–Pituitary–Adrenal Axis Activity and the Remission Status of Major Depression: Effects on Functionality and Quality of Life
Source: Brain Sci. 2021 Apr 13;11(4):495. doi: 10.3390/brainsci11040495 (PMC8069655; doi:10.3390/brainsci11040495)
Supplement: Supplementary file 1 [file brainsci-11-00495-s001.zip › TableS6.pdf]

Table S6. Results of multiple linear regression analyses exploring the association of childhood maltreatment, HPA axis function, and MDD remission status with quality of life (EQ-5D – Index scores) in MDD patients (n=97).

	Model 1 $R^2 = 0.114$		Model 2 $R^2 = 0.565$		Model 3 $R^2 = 0.642$		Model 4 $R^2 = 0.676$		Final model $R^2 = 0.715$	
	β	p	β	p	β	p	β	p	β	P
CTQ- emotional abuse	-0.168	0.411	0.083	0.591	0.077	0.659	0.134	0.481	0.046	0.802
CTQ- physical abuse	-0.042	0.808	-0.191	0.144	-0.187	0.218	-0.255	0.119	-0.230	0.139
CTQ- sexual abuse	-0.032	0.829	-0.108	0.314	-0.128	0.266	-0.176	0.156	-0.163	0.169
CTQ- emotional neglect	-0.199	0.261	0.078	0.564	0.048	0.729	0.051	0.734	0.026	0.859
CTQ- physical neglect	0.036	0.823	-0.089	0.449	-0.058	0.642	-0.099	0.449	-0.074	0.554
STAI - state			-0.328	0.021	-0.200	0.207	-0.227	0.162	-0.228	0.141
STAI - trait			-0.489	0.003	-0.455	0.011	-0.371	0.057	-0.471	0.016
Gender					0.081	0.484	0.053	0.654	0.089	0.439
Age					-0.090	0.531	-0.119	0.411	-0.175	0.215
Years of education					0.037	0.761	0.002	0.989	0.047	0.706
BMI					-0.034	0.769	-0.062	0.637	-0.052	0.679
Tobacco consumption (cig/day)					-0.154	0.186	-0.188	0.128	-0.251	0.041
Remitted MDD					0.297	0.042	0.338	0.026	0.209	0.177
PSQI					0.043	0.774	0.055	0.736	0.072	0.643
Waking cortisol							0.156	0.249	0.108	0.409
CAR							0.053	0.717	-0.208	0.268
DSTR							-0.056	0.641	-0.038	0.740
Diurnal cortisol slope							0.157	0.202	0.175	0.138
CAR x Remitted MDD									0.356	0.039

EQ-5D – Index score was considered as the dependent variable.

β: standardized beta coefficient.

Abbreviations: MDD, major depressive disorder; EQ-5D, Euro Quality of Life 5-dimensions questionnaire; CTQ, Childhood Trauma Questionnaire; STAI, State-Trait Anxiety Inventory; BMI, body mass index; PSQI, Pittsburgh Sleep Quality Index; CAR, cortisol awakening response calculated to the increase in cortisol; DSTR, dexamethasone suppression test ratio.

Analyses performed using transformed cortisol variables, outliers excluded.

Non-significant interaction terms were excluded in the final equation.
