# Supplementary material for: Childhood Maltreatment and Its Interaction with Hypothalamic–Pituitary–Adrenal Axis Activity and the Remission Status of Major Depression: Effects on Functionality and Quality of Life
Source: Brain Sci. 2021 Apr 13;11(4):495. doi: 10.3390/brainsci11040495 (PMC8069655; doi:10.3390/brainsci11040495)
Supplement: Supplementary file 1 [file brainsci-11-00495-s001.zip › TableS7.pdf]

Table S7. Results of multiple linear regression analyses exploring the association of childhood maltreatment, HPA axis function, and MDD remission status with quality of life (EQ-VAS) in MDD patients (n=97).

	Model 1 $R^2 = 0.164$		Model 2 $R^2 = 0.675$		Model 3 $R^2 = 0.743$		Final model 4 $R^2 = 0.805$	
	β	p	β	p	β	p	β	p
CTQ- emotional abuse	-0.389	0.066	-0.140	0.322	-0.131	0.445	-0.222	0.198
CTQ- physical abuse	0.291	0.101	0.147	0.211	0.136	0.345	0.166	0.236
CTQ- sexual abuse	0.072	0.628	-0.012	0.900	-0.019	0.851	-0.016	0.872
CTQ- emotional neglect	-0.274	0.121	-0.010	0.935	-0.023	0.851	-0.057	0.641
CTQ- physical neglect	0.197	0.228	0.074	0.484	0.090	0.426	0.063	0.552
STAI - state			-0.472	<0.001	-0.311	0.029	-0.365	0.008
STAI - trait			-0.383	0.008	-0.332	0.031	-0.170	0.283
Gender					0.098	0.332	0.083	0.380
Age					-0.068	0.607	-0.170	0.196
Years of education					-0.025	0.810	-0.089	0.385
BMI					-0.109	0.289	-0.129	0.218
Tobacco consumption (cig/day)					-0.125	0.216	-0.129	0.188
Remitted MDD					0.211	0.111	0.236	0.057
PSQI					-0.086	0.512	-0.145	0.292
Waking cortisol							0.067	0.511
CAR							-0.089	0.459
DSTR							0.036	0.713
Diurnal cortisol slope							0.244	0.014

EQ-5D – Index score was considered as the dependent variable.

β: standardized beta coefficient.

Abbreviations: MDD, major depressive disorder; EQ-5D, Euro Quality of Life 5-dimensions questionnaire; VAS: visual analogue scale; CTQ, Childhood Trauma Questionnaire; STAI, State-Trait Anxiety Inventory; BMI, body mass index; PSQI, Pittsburgh Sleep Quality Index; CAR, cortisol awakening response calculated to the increase in cortisol; DSTR, dexamethasone suppression test ratio.

Analyses performed using transformed cortisol variables, outliers excluded.

No significant interaction terms between HPA axis measures, CTQ variables or depression remission status were found in the analysis.
